# Global trends of traditional Chinese exercises for musculoskeletal disorders treatment research from 2000 to 2022: A bibliometric analysis

**DOI:** 10.3389/fnins.2023.1096789

**Published:** 2023-02-10

**Authors:** Chong Guan, Yuanjia Gu, Ziji Cheng, Fangfang Xie, Fei Yao

**Affiliations:** Shanghai Municipal Hospital of Traditional Chinese Medicine, Shanghai University of Traditional Chinese Medicine, Shanghai, China

**Keywords:** traditional Chinese exercises, musculoskeletal disorders, bibliometric, VOSviewer, CiteSpace

## Abstract

**Background:**

Traditional Chinese exercise has been shown to be effective in relieving long-term chronic pain, physical dysfunction, decreased ability to participate in society and decreased quality of life in musculoskeletal diseases. In recent years, there has been a steady increase in publications on the treatment of musculoskeletal disorders by traditional Chinese exercises. The purpose of this study is to review the characteristics and trends of Chinese traditional exercise studies on musculoskeletal diseases published since 2000 through bibliometric analysis, and identify current research hotspots, so as to guide the direction of future research.

**Methods:**

Publications regarding traditional Chinese exercises for musculoskeletal disorders from 2000 to 2022 were downloaded from the Web of Science Core Collection. VOSviewer 1.6.18 and CiteSpace V software were used for bibliometric analyses. Bibliometric visualization and comparative analysis were conducted for authors, cited authors, journals, co-cited journals, institutions, countries, references, and keywords.

**Results:**

A total of 432 articles were obtained, with an upward trend over time. The most productive countries and institutions in this field are the USA (183) and Harvard University (70). Evidence-based Complementary and Alternative Medicine (20) was the most prolific journal, Cochrane Database System Review (758) was the most commonly cited journal. Wang Chenchen published the largest number of articles (18). According to high frequency keywords, the hot spot musculoskeletal disorder and the type of traditional Chinese exercise are knee osteoarthritis and Tai Chi.

**Conclusion:**

This study provides a scientific perspective for the research of traditional Chinese exercises for musculoskeletal disorders and provides valuable information for researchers to discover the current research status, hot spots and new trends of future research.

## Background

Musculoskeletal disorders are a group of diseases of the body’s motor structures, particularly the bones, joints, muscles, fascia, and other supporting structures such as ligaments and cartilages (Safiri et al., 2021). Musculoskeletal disorders include neck pain, low back pain, osteoarthritis, rheumatoid arthritis and other diseases (Jin et al., 2020). Many musculoskeletal disorders are recurrent or lifelong, with the main consequences are long-term chronic pain, physical dysfunction, self-care ability, social participation and reduced quality of life (Tung et al., 2021). Patients with musculoskeletal disorders are more likely to suffer from depression, anxiety, and further exacerbation of musculoskeletal disorders (Singh, 2022). Long-term musculoskeletal disorders may even increase the incidence of accidental disability and death, especially in the elderly. According to the WHO, falls are the leading cause of injury and death among older adults which also the second leading cause of unintentional injury deaths worldwide. Musculoskeletal disorders such as muscle weakness, knee osteoarthritis, and fractures are risk factors for falls (Dunlop et al., 2014; Wang et al., 2022b). Epidemiological studies in many developed countries show that musculoskeletal disorders are characterized by high rates of disability and absenteeism (Shanahan, 2019). It has a serious negative impact on the social life and emotions of patients, especially elderly patients. It causes great trouble to human health and great economic burden to individuals, families and even the society (Wu et al., 2021). Studies have shown that obesity, poor nutrition, smoking habits, excessive exercise and occupational injuries are all risk factors for musculoskeletal disorders.

Adjusting diet, moderate exercise, quitting smoking and drinking, are considered to be effective ways to reduce the incidence of muscle diseases and treat musculoskeletal diseases (Gwinnutt et al., 2022a,b). Traditional Chinese exercises is a kind of physical and mental exercise and complementary medical exercise therapy with Chinese cultural characteristics (Guo et al., 2016). Common traditional Chinese exercises include Tai Chi, Yi Jin Jing, Baduanjin, Wuqinxi and so on (Feng et al., 2020). Traditional Chinese exercises are low-intensity aerobic exercise suitable for people of all ages due to its slow and gentle movements. For example, Tai Chi is a traditional Chinese exercise which is widely regarded as a physical and mental exercise (Zou et al., 2018). Tai Chi is widely practiced for its health benefits (Lan et al., 2013). Tai Chi is a low intensity, non-competitive and non-impact exercise. It puts emphasis on the coordination of breathing, thinking and physical activity. The traditional Chinese exercises are gradually formed on the basis of the holistic concept of Traditional Chinese medicine, with the theory of five elements, Yin and Yang, meridians and zangfu. Many studies have shown that Tai Chi is beneficial for the medical management of musculoskeletal disorders such as neck pain, low back pain, knee osteoarthritis, Fibromyalgia and so on (Zhang et al., 2017; Slomski, 2018; Xie et al., 2021; Wang et al., 2022a.

Bibliometrics is widely used to discover research hotspots and analyze research results and research trends, which can help researchers identify current research hotspots and guide future research directions. In recent years, bibliometrics has been applied to Traditional Chinese exercises research fields, such as Tai Chi and traditional Chinese exercises for pain so on (Zhang et al., 2020a; You et al., 2021). But no article has ever been written to analyze the treatment of musculoskeletal disorders by traditional Chinese exercises from a bibliometric perspective.

Therefore, from the perspective of bibliometrics, this article sorts out and summarize the development, research hotspots and development trends of traditional Chinese exercises for musculoskeletal research. The aim of this study is to provide an overview of the published characteristics and trends of traditional Chinese exercises for musculoskeletal disorders since 2000 through bibliometric analysis.

## Materials and methods

### Data sources and search strategy

We selected Web of Science Core Collection (WoSCC) as the data source to identify and extract relevant publications. To cover as many target documents as possible, we chose terms that most scientific publications might use to build the search strategy. Terms related to traditional Chinese exercises and musculoskeletal disorders were extracted from the Medical Subject Headings (MeSH) in PubMed (Supplementary material for retrieval information). In order to avoid possible problems such as duplication, missing or inconsistency with the theme, it is necessary to screen and standardize the data before analysis to avoid the quality of the data itself affecting the results. The detailed search strategy is in Supplementary material.

The inclusion criteria are as follows:(1) The literature retrieval period is from 1 January 2000 to 14 August 2022; (2) the literature type is “article” and “review;” and (3) the language type is English.

The exclusion criteria are as follows: (1) The literature whose research topic was not related to the traditional Chinese exercise exercises for musculoskeletal diseases; (2) Letters, reports, short papers or briefs.

**FIGURE 1:**
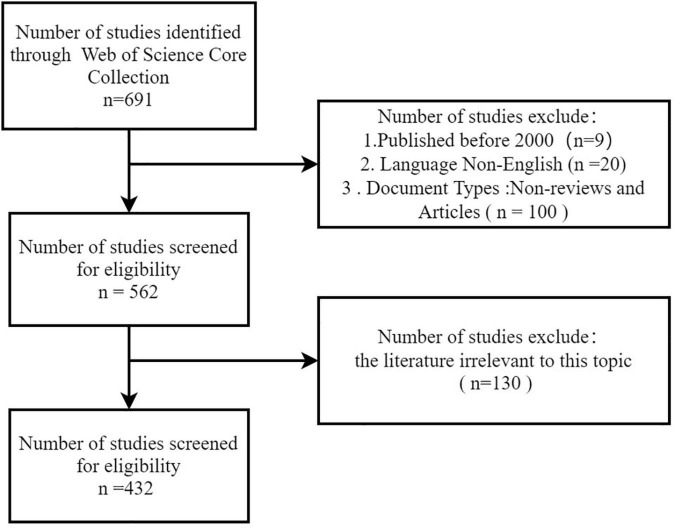
Flow diagram of literature search and screening.

The errata type of documents resulted in 432 papers. The data collection flow chart is shown in Figure 1.

The steps of data review and screening in this study were as follows: (1) Two team members independently reviewed the articles and screened out the articles which were inconsistent with the research topic. These controversial articles were voted out by our team. (2) We corrected and harmonized of the selected articles, institutions and countries in order to avoid the influence of author, institution, country name on the results. (3) Keywords were standardized, because the non-standardized keywords would lead to meaningless repetition in the keyword co-occurrence graph due to the inconsistency of pos and plural and singular versions, so keywords were standardized.

For example, the three keywords of “metaanalysis,” “meta analysis,” and “Meta analysis” are unified as “meta-analysis” in this study.

### Statistical analysis

VOSviewer 1.6.18 and CiteSpace 6.1.R1 software were used to conduct bibliometrics analysis on key characteristics of literatures such as the number of publications, countries/regions, institutions, authors, journals, literatures, keywords and so on. VOSviewer and CiteSpace are widely used bibliometric tools. VOSviewer is a freely available program which has the added advantage of graphical representation in bibliometric mapping by displaying large bibliometric maps in an easy-to-interpret manner. CiteSpace is a free and available program, which has greater advantages in intuitively catching up the research hotspots and evolutionary processes of various fields in the knowledge system and predicting the development trends of various fields.

## Results

### Analysis of annual publications

**FIGURE 2:**
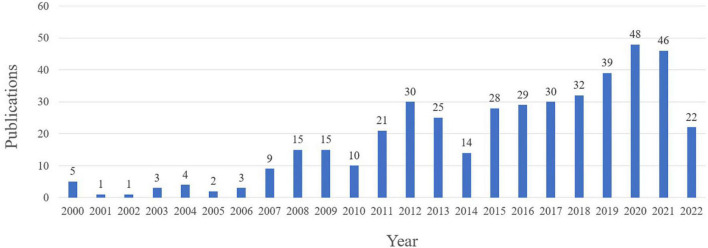
The number of annual publications on traditional Chinese exercises for musculoskeletal disorders research from 2000 to 2022.

Figure 2 shows the number of published in the field of traditional Chinese exercises for musculoskeletal disorders. From 2000 to 2006, there were few publications on the treatment of musculoskeletal disorders by traditional Chinese exercises. The number of articles was no more than five which indicated that this research field has not received attention. The number of publications from 2007 to 2014 was significantly higher than that before 2006, but the number of publications during the period did not change much, indicating that this field has gradually attracted attention. From 2014 to 2021, the number of publications was increased. Although the number of documents issued will decrease after 2020, this may be because the arrival of COVID-19 has hindered the traditional Chinese exercises, which is mainly clinical research. This trend is consistent with what has been observed in other areas of research. From the trend of Figure 2, It is found that more research on traditional Chinese exercises for musculoskeletal disorders is being conducted.

### Analysis of journal

A total of 432 publications related to Chinese traditional exercise for musculoskeletal disorders were published in 209 journals. Table 1 shows the top 10 journals in terms of number of publications. Most of the publishers of these journals are located in the USA or England. The top three journals in terms of the number of publications are Evidence Based Comprehensive and Alternative Medicine, Medicine and Journal of Alternative and Comprehensive Medicine.

**TABLE 1:** Top 10 journals with the highest frequency with traditional Chinese exercises for musculoskeletal disorders.

Rank	Journal	Publications	Country	Citations (WoS)	Average citation/publication	Impact factor (2021)	Categories	Quartile (JCR)	OA
1	Evidence-Based Complementary and Alternative Medicine	20	England	342	17.10	2.650	Integrative and complementary medicine	Q3	Yes
2	Medicine	18	USA	38	2.11	1.817	Medicine, general, and internal	Q3	Yes
3	Journal of Alternative and Complementary Medicine	17	USA	390	22.94	2.381	Integrative and complementary medicine	Q3	No
4	Complementary Therapies in Medicine	10	England	172	17.20	3.335	Integrative and complementary medicine	Q2	Yes
5	Bmc Musculoskeletal Disorders	7	England	185	26.43	2.562	Orthopedics rheumatology	Q3; Q4	Yes
5	Cochrane Database of Systematic Review	7	England	767	109.57	11.874	Medicine, general, and internal	Q1	No
5	Osteoarthritis and Cartilage	7	England	245	35.00	7.507	Orthopedics rheumatology	Q1; Q1	No
5	Pain Medicine	7	USA	245	35.00	3.637	Anesthesiology medicine, general, and internal	Q2; Q2	No
5	Trials	7	England	56	8.00	2.728	Medicine, general, and internal	Q4	Yes
10	Arthritis Care and Research	6	USA	2,132	355.33	5.178	Rheumatology	Q2	No
10	British Journal of Sports Medicine	6	England	657	109.50	18.479	Sport sciences	Q1	No
10	Current Pain and Headache Reports	6	USA	290	48.33	3.904	Clinical neurology	Q2	No
10	Journal of Physical Therapy Science	6	Japan	92	15.33	0.392	Rehabilitation	Q4	No
10	PLoS One	6	USA	333	55.50	3.752	Multidisciplinary sciences	Q2	Yes

The analysis of co-citation journals shows that important knowledge sources are distributed in a specific field. Our statistical analysis results show that 4,113 articles published in Chinese traditional exercise for musculoskeletal disorders have been co-cited.

Table 2 shows the top 10 cited journals in terms of number of publications. The most frequently cited journal is Cochrane Database of Systematic Reviews, followed by Journal of Rheumatology and Pain. We found that most of the top 10 journals cited were in Q1, indicating that the research in this field mainly cited high-quality journals. According to the citation analysis of journals, the most cited journal is Annals of Internal Medicine, a high-level journal in the medical field, with a total of six articles, a single article was cited 75.83 times. This shows that the quality of the articles published in this journal is high and have received extensive attention in this research field.

**TABLE 2:** Top 10 cited journals with the highest frequency with traditional Chinese exercises for musculoskeletal disorders.

Rank	Journal	Citations	Country	IF (2021)	Categories	Quartile (JCR)
1	Cochrane Database of Systematic Reviews	758	England	11.874	Medicine, general and internal	Q1
2	Journal of Rheumatology	556	Canada	5.346	Rheumatology	Q2
3	Pain	502	USA	7.926	Neurosciences clinical neurology anesthesiology	Q1; Q1; Q1
4	Arthritis & Rheumatism-Arthritis Care & Research (Arthritis and Rheumatism)	489	USA	/	Rheumatology	/
5	Archives of Physical Medicine and Rehabilitation	453	USA	4.06	Rehabilitation sport sciences	Q1; Q1
6	Journal of Alternative and Complementary Medicine	408	USA	2.381	Integrative and complementary medicine	Q3
7	Spine	402	USA	3.269	Orthopedics clinical neurology	Q3; Q2
8	Journal of the American Geriatrics Society	392	USA	7.538	Gerontology geriatrics and gerontology	Q1; Q1
9	Annals of Internal Medicine	329	USA	51.598	Medicine, general, and internal	Q1
10	Osteoarthritis and Cartilage	327	England	7.507	Orthopedics rheumatology	Q1; Q1

**FIGURE 3:**
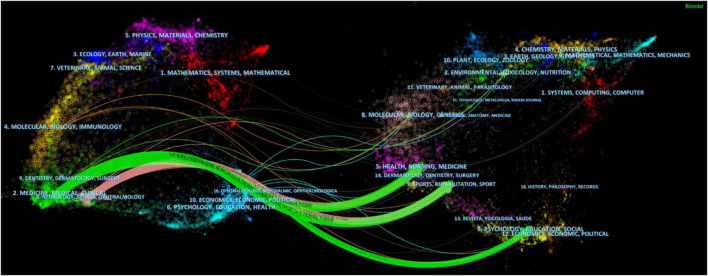
The dual-map overlay of journals.

Figure 3 is a dual-map superposition of the references in traditional Chinese exercises for musculoskeletal disorders research. Cited journals are on the left and co-cited journals are on the right. The reference link comes from the journal on the left side of the map and points to the journal on the right side of the map. The green path indicates that documents published in “medicine, medical clinical” journals are often cited by “health, nursing, medicine, sports, rehabilitation, sport” and “psychology, education, social” journals. Pink path indicates that documents published in journals are often cited by “health, nursing, medicine, sports, rehabilitation, sport” journals.

### Analysis of country and institutions

VOSviewer is used to generate a network visualization map. In order to make the network clear, 16 countries/regions that publish at least five articles are visualized. The size of the node is determined by the number of published articles (the more the number, the larger the node). The lines between nodes represent the cooperation between countries/regions (the stronger the cooperation, the wider the lines). The number of total link strength reflects the strength of cooperation between countries/regions. From 2000 to 2022, articles on traditional Chinese exercises for musculoskeletal disorders came from 38 different countries/regions independently or cooperatively. Table 3 shows top 10 countries for publications of traditional Chinese medicine in musculoskeletal disorder. The publications authors are mainly from the USA, with 183 articles published, followed by China (129). Canada (41), Australia (31), and Germany (25) also made considerable contributions to the research of Chinese traditional exercise for musculoskeletal disorders.

**TABLE 3:** Top 10 countries for publications of traditional Chinese exercises for musculoskeletal disorders.

Rank	Country	Publications	Citations	Average citations/publication
1	USA	183	9,308	50.86
2	China	129	2,151	16.67
3	Canada	41	4,602	112.24
4	Australia	31	1,761	56.81
5	Germany	25	828	33.12
6	England	23	1,129	49.09
7	South Korea	16	568	35.5
8	Italy	12	107	8.92
9	Spain	11	222	20.18
9	Sweden	11	422	38.36

**FIGURE 4:**
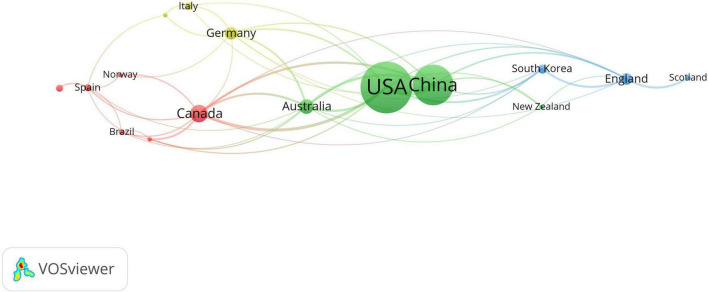
Map of countries with publications in traditional Chinese exercises for musculoskeletal disorders research from 2000 to 2022.

Table 4 shows top 10 institutions with the highest frequency of traditional Chinese exercises for musculoskeletal disorders. In order to make the network clear, 36 institutions that publish at least five articles are visualized (Figure 4). The top 10 institutions with the largest number of publications have been identified, including 4 from China, 3 from the USA, 2 from Australia, 1 from Canada and 1 from Germany. The distribution of the top 10 organizations in terms of the number of documents issued is consistent with the distribution characteristics of each country. Among them, Harvard University contributed 31 articles to the largest number of publications in the USA. Shanghai University of Traditional Chinese Medicine contributed 17 articles to the largest number of publications in China. From the time line of articles published by institutions generated by VOSviewer, around 2020, university of traditional Chinese medicine, especially Shanghai University of Traditional Chinese Medicine and Chengdu University of Traditional Chinese Medicine paid more attention to the research of traditional Chinese exercises on musculoskeletal treatment.

**TABLE 4:** Top 10 Institutions with the highest frequency of traditional Chinese exercises for musculoskeletal disorders.

Rank	Country	Publications	Citations	Average citations/publication
1	Harvard University	31	972	31.35
2	Tufts University	22	1,616	73.45
3	Shanghai University of Traditional Chinese Medicine	17	139	8.18
4	Shanghai University of Sport	15	255	17.00
5	University of Ottawa	13	2,077	159.77
6	The Chinese University of Hong Kong	12	456	38.00
7	University of Duisburg-Essen	12	427	35.58
8	Fujian University of Traditional Chinese Medicine	11	161	14.64
9	North Carolina University	11	970	88.18
10	The University of Sydney	10	961	96.10
10	University of Toronto	10	1,180	118.00

### Analysis of authors

Table 5 shows the top ten authors in this field. Among the authors in the field of traditional Chinese exercises for musculoskeletal disorders, Wang CC published the most articles (Zhang et al., 2017), which were cited 946 times, with an average of 52.56 times. Three of the top five articles cited in total are published by Wang CC. The top five active authors all published at least 10 articles. Mcalindon Timothy is the researcher who has cited the most per article, with an average of 93.86 times for each article.

**TABLE 5:** Top 10 authors for publications on traditional Chinese exercises for musculoskeletal disorders.

Rank	Author	Publications	Citations	Average citations/publication
1	Wang, Chenchen	18	946	52.56
2	Harvey, William F	11	356	32.36
3	Driban, Jeffrey B	10	401	40.10
3	Price, Lori Lyn	10	401	40.10
3	Zou, Liye	10	330	33.00
6	Wayne, Peter M	9	248	27.56
7	Dobos, Gustav	8	290	36.25
8	Cramer, Holger	7	138	19.71
8	Mcalindon, Timothy	7	657	93.86
8	Rones, Ramel	7	655	93.57

### Analysis of cited reference and co-cited reference

Table 6 shows the most cited articles in the field of traditional Chinese exercises for musculoskeletal disorders. Among the top 10 most cited publications on traditional Chinese exercises for musculoskeletal disorders, there was five systematic reviews, three guideline literature, one clinical randomized controlled trial and one review. The results show that high quality systematic review can sort out the evidence of clinical research in this field and get higher level evidence.

**TABLE 6:** The top 10 references based on the number of citations traditional Chinese exercises for musculoskeletal disorders.

Rank	Citations		First author	Corresponding author	Document type	Year	Journal	IF (2021)
1	1,817	American College of Rheumatology 2012 recommendations for the use of non-pharmacologic and pharmacologic therapies in osteoarthritis of the hand, hip, and knee	Hochberg, Marc C.	Hochberg, Marc C	Article	2012	Arthritis Care and Research	5.178
2	911	Non-invasive treatments for acute, subacute, and chronic low back pain: A clinical practice guideline from the American College of Physicians	Qaseem, Amir	Qaseem, Amir	Article	2017	Annals of Internal Medicine	51.598
3	626	2019 American College of Rheumatology/Arthritis Foundation Guideline for the Management of Osteoarthritis of the Hand, Hip, and Knee	Kolasinski, Sharon L.	Kolasinski, Sharon L	Article	2020	Arthritis and Rheumatology	15.483
4	341	Non-pharmacologic therapies for low back pain: A systematic review for an American College of Physicians Clinical Practice Guideline	Chou, Roger	Chou, Roger	Review	2017	Annals of Internal Medicine	51.598
5	308	Exercise for osteoarthritis of the knee: A Cochrane systematic review	Fransen, Marlene	Van der Esch, Martin	Review	2015	British Journal of Sports Medicine	18.479
6	299	The effect of Tai Chi on health outcomes in patients with chronic conditions–A systematic review	Wang, CC	Wang, CC	Review	2004	Archives of Internal Medicine	/
7	260	Effects of Tai Chi exercise on pain, balance, muscle strength, and perceived difficulties in physical functioning in older women with osteoarthritis: A randomized clinical trial	Song, R	Bae, SC	Article	2003	Journal of Rheumatology	5.346
8	255	Physical activity and exercise for chronic pain in adults: An overview of cochrane reviews	Geneen, Louise J	Geneen, Louise J.	Review	2017	Cochrane Database of Systematic Reviews	11.874
9	247	A review of the clinical evidence for exercise in osteoarthritis of the hip and knee	Bennell, Kim L.	Bennell, Kim L.	Review	2011	Journal of Science and Medicine in Sport	4.597
10	209	Tai Chi: Physiological characteristics and beneficial effects on health	Li, JX	Li, JX	Review	2001	British Journal of Sports Medicine	18.479

Co-citation means that two articles appear in the citation of the third cited article at the same time, thus forming a co-citation relationship. Co-citation indicates that the cited literature is related to the corresponding research in terms of content and the literature usually contains high-quality content with significant influence in a specific research field. In addition, the relationship between literature co-citations may change over time. The study of the influence of network on literature co-citation can facilitate the investigation of the development and evolution of specific disciplines.

Table 7 shows the most frequently cited articles published in the field of traditional exercise exercises for musculoskeletal disorders. Among the top 10 most cited publications on traditional Chinese exercises for musculoskeletal disorders, there were seven clinical randomized controlled trials, two reviews, one guideline literature and one systematic review. The results show that high quality clinical research is the focus of researchers in this field, which is helpful for the research in the field of traditional Chinese exercises for musculoskeletal disorders.

**TABLE 7:** The top 10 co-cited references based on the number of citations traditional Chinese exercises for musculoskeletal disorders.

Rank	Citations	Title	First author	Corresponding author	Document type	Year	Journal	IF (2021)
1	91	Tai Chi is effective in treating knee osteoarthritis: A randomized controlled trial	Wang, CC	Wang, CC	Article	2009	Arthritis and Rheumatism	/
2	82	Effects of Tai Chi exercise on pain, balance, muscle strength, and perceived difficulties in physical functioning in older women with osteoarthritis: A randomized clinical trial	Song, R	Bae, SC	Article	2003	Journal of Rheumatology	5.346
3	67	Physical activity for osteoarthritis management: A randomized controlled clinical trial evaluating hydrotherapy or Tai Chi classes	Fransen, Marlene	Fransen, Marlene	Article	2007	Arthritis and Rheumatism	/
4	65	A randomized trial of Tai Chi for fibromyalgia	Wang, CC	Wang, CC	Article	2010	New England Journal of Medicine	176.082
5	63	Group and home-based Tai Chi in elderly subjects with knee osteoarthritis: A randomized controlled trial	Brismee, Jean-Michel	Shen, Chwan-Li	Article	2007	Clinical Rehabilitation	2.884
6	61	The effect of Tai Chi on health outcomes in patients with chronic conditions–A systematic review	Wang, CC	Wang, CC	Review	2004	Archives of Internal Medicine	/
7	51	Effects of t’ai chi training on function and quality of life indicators in older adults with osteoarthritis	Hartman, CA	Hartman, CA	Article	2000	Journal of The American Geriatrics Society	7.538
8	49	Tai chi Qigong for the quality of life of patients with knee osteoarthritis: A pilot, randomized, waiting list controlled trial	Lee, HJ	Lee, HJ	Article	2009	Clinical Rehabilitation	2.884
9	34	Development of criteria for the classification and reporting of osteoarthritis. Classification of osteoarthritis of the knee. Diagnostic and therapeutic criteria committee of the American rheumatism association	R. Altman	R. Altman	Review	1986	Arthritis and Rrheumatism	/
10	33	Validation study of WOMAC: a health status instrument for measuring clinically important patient relevant outcomes to antirheumatic drug therapy in patients with osteoarthritis of the hip or knee	N Bellamy	N Bellamy	Review	1988	Journal of Rrheumatology	5.346
10	33	Challenges inherent to t’ai chi research: Part I–t’ai chi as a complex multicomponent intervention	Wayne, PM	Wayne	Review	2008	Journal of Alternative and Complementary Medicine	2.381

### Analysis of co-occurrence keyword

Keywords with high frequency can reveal hot spots in past research and keywords with strong burst power can predict future research frontiers. By August 2022, the top 10 keywords of frequency were Tai Chi, Exercise, Pain, Older Adult, Randomized Controlled Trial, Osteoarthritis, Quality of Life, Knee Osteoarthritis, Management and Low Back Pain (Table 8). According to the high-frequency keywords, we can infer that knee osteoarthritis is the most frequently studied musculoskeletal disorder in this field, and Tai Chi is the most frequently studied traditional Chinese exercises in this field.

**TABLE 8:** Top 20 keywords in the treatment of traditional Chinese exercises for musculoskeletal disorders.

Rank	Keyword	Frequence
1	Tai chi	231
2	Exercise	152
3	Pain	112
4	Older adult	105
5	Randomized controlled trial	103
6	Osteoarthritis	85
6	Quality of life	85
8	Knee osteoarthritis	80
9	Management	80
10	Low back pain	71
11	Physical activity	58
12	Meta analysis	53
13	Balance	51
14	Rheumatoid arthritis	50
15	Health	49
16	Qigong	48
17	Clinical trials	46
18	Efficacy	43
19	Fibromyalgia	42
20	Arthritis	39

**FIGURE 5:**
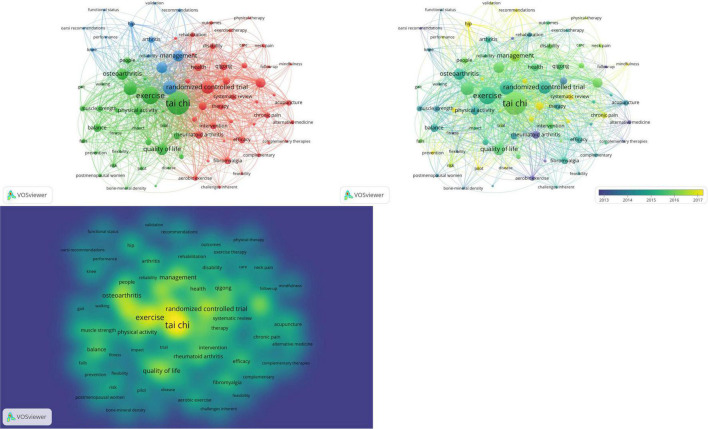
Co-occurrence network of high-frequency keywords of traditional Chinese exercises for musculoskeletal disorders.

In this study, 432 articles were merged and classified by VOSviewer, 83 keywords that were used more than 10 times were clustered and analyzed. The results are shown in Figure 5 and Table 9. In the VOSviewer keyword network visualization, 83 keywords are grouped into clusters and different clusters are labeled with different colors. As shown in Figure 5, The clusters of red, green and blue were found. The red and green clusters are relatively independent, while the blue clusters are associated with both red and green clusters. The main keywords in the red cluster are Randomized Controlled Trial; Low Back Pain; Meta Analysis; Rheumatoid Arthritis; The main keywords of green cluster Tai Chi; Exercise; Older Adult; Osteoarthritis; Quality of Life. The main keyword of blue cluster is Pain; Knee Osteoarthritis; Management.

**TABLE 9:** Cluster of keywords in the traditional Chinese exercises for musculoskeletal disorders.

Cluster	Color	Keywords
1	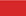	Randomized controlled trial; Low back pain; meta analysis; rheumatoid arthritis; health; qigong; efficacy; fibromyalgia; intervention; therapy; systematic review; chronic pain; disability; prevalence; acupuncture; aerobic exercise; toga; quality; alternative medicine; complementary; outcomes; depression; neck pain; exercise therapy; recommendations; double-blind; care; complementary and alternative medicine; controlled-trial; challenges inherent; cognitive-behavioral therapy; follow-up; mindfulness meditation; self-efficacy; symptoms; complementary therapies; feasibility; mindfulness; physical-therapy; self-management
2	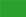	Tai chi; exercise; older adult; osteoarthritis; quality of life; physical activity; balance; people; muscle strength; program; women; strength; adult; physical function; risk; falls; pilot; prevention; trial; postmenopausal women; impact; risk factors; walking; bone-mineral density; gait; classification; disease; flexibility; fitness; individuals
3	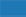	Pain; knee osteoarthritis; management; clinical trials; arthritis; hip; rehabilitation; knee; reliability; oarsi recommendations; performance; functional status; validation

The VOSviewer keyword symbiosis visualization hotspot map can identify hotspots in the field of traditional Chinese exercises for musculoskeletal research where most of the research was conducted. Keywords were clustered in the heat map, keywords with different frequencies presented different colors, such as the high density (red) among the above keywords.

### Analysis of keyword burst

**FIGURE 6:**
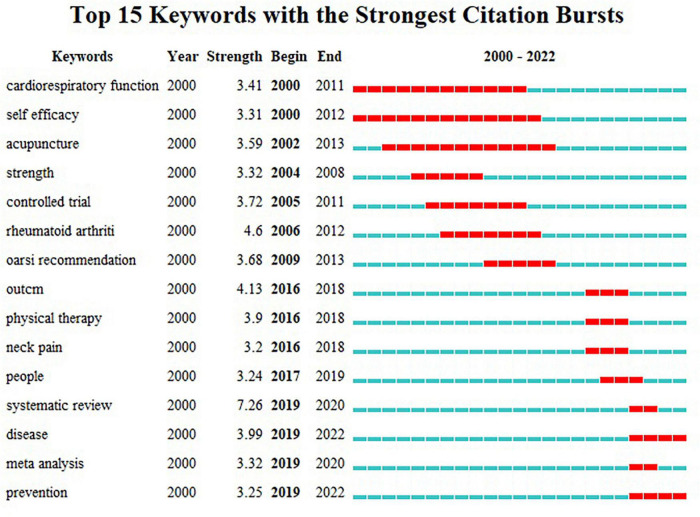
Top 15 keywords with the strongest citation bursts.

Keyword burst is a valuable indicator reflecting the most active research topic in this field. It can not only reflect research hotspots in this field, but also reflect changes in hotspots and predict future research trends. As shown in Figure 6, the top 15 keywords with the strongest strength of burst are presented. The changes in keyword outbreaks over the past 22 years have proved the evolution of the hotspots of traditional Chinese exercises for musculoskeletal disorders. The keyword “cardiorespiratory function” had a strength of 3.41 from 2000 to 2011. The keyword “self-efficiency” has the longest cycle, which is 12 years and the strength is 3.31. The remaining eight keywords which have received more attention since 2016 are the focus of current research in this field. The keywords of explosion include “outcome” (strength = 4.13), “physical therapy” (strength = 3.9), “neck pain” (strength = 3.2), “people” (strength = 3.24), “systematic review” (strength = 7.26), “disease” (strength = 3.99), “meta-analysis” (strength = 3.32) and “prevention” (strength = 3.25).

## Discussion

### General information

From 2000 to 2022, the number of publications on traditional Chinese exercises for musculoskeletal disorders showed an increasing trend. This research field has attracted more and more scholars’ attention and become a new hot field. This phenomenon may be related to the clinical effect of Chinese traditional exercise as complementary alternative medicine and sports medicine in the treatment of musculoskeletal disorders.

The top 10 journals published 130 articles, accounting for 30.09% of the total number of articles published, indicating that the top 10 academic journals have a strong interest in the articles on musculoskeletal research of traditional Chinese exercises. Among them, Medicine, evidence-based complementary and alternative medicine and other journals belong to OA journals, indicating that the strong development of open-access journals in recent years has greatly promoted the research progress in this field. Most of the journals belong to the category of complementary and alternative medicine, indicating that the field of complementary and alternative medicine is more interested in the musculoskeletal disorders research of traditional Chinese exercises.

The USA is the first country to study this field. Meanwhile, two of the top five institutions are from the USA, led by Harvard University. All these indicate that the USA is the most influential country in the field of traditional Chinese exercises treatment of musculoskeletal disorders. In recent years, China’s influence in this field has gradually increased and there is a good development trend in this field in the future. Two of the top five are from China, with Shanghai University of Traditional Chinese Medicine and Shanghai University of Physical Education published 17 and 15 papers respectively. In addition to China and the USA, many countries have carried out research in this field, indicating that research attention in this field has increased in recent years. Therefore, there should be a stronger network of collaboration between more countries, institutions and authors, especially in China.

Although the traditional Chinese exercises originated in China, there are more than 20 universities of traditional Chinese exercises for China, which do not publish as many publications as other institutions. This may be related to the fact that most Chinese physicians pay more attention to the cultural attributes of traditional Chinese exercises. Interestingly, universities of traditional Chinese medicine are increasingly aware of this phenomenon and are gradually devoting themselves to the research of traditional Chinese exercises for musculoskeletal disorders.

According to the keyword frequency, we found that Tai Chi is the most commonly used traditional Chinese exercise for musculoskeletal diseases, which we believe may be related to the following reasons: (1) Tai Chi has been practiced in China for centuries. At present, Tai Chi has about 150 million practitioners in more than 150 countries and regions around the world. It is the most widely spread traditional Chinese exercises; (2) There are large body of evidence on the health effects of Tai chi, with more than 500 articles and 120 systematic reviews of the health benefits of Tai chi published. Based on such evidence-based advice, more researchers will focus on Tai Chi in their research; (3) Modern researchers have developed many simplified forms of Tai Chi, which shorten the learning time of practitioners and promote the spread of Tai Chi.

### Global trend and research hotspots

Frequently keywords and burst keywords are the core content of the research literature topic. Cluster analysis was carried out based on keywords and finally three colors clusters were formed. Then, according to the keyword burst analysis, the research hotspots and development frontiers in the field are identified. The main contents are as follows: The efficacy evaluation of traditional Chinese exercises for the treatment of musculoskeletal disorders; the efficacy of traditional Chinese exercises for the treatment of musculoskeletal disorders dysfunctions; the pain improvement of traditional Chinese exercises for musculoskeletal disorders.

Cluster one keywords in this cluster can be divided into two categories. One focuses on research methods, such as randomized controlled trial, systematic review, and Meta-analysis. This cluster focused on the evaluation of the efficacy of traditional Chinese exercises for musculoskeletal disorders. The other type focuses on clinical diseases such as low back pain, rheumatoid arthritis, fibromyalgia and neck pain. In early studies, researchers focused on the therapeutic effects of Tai Chi on knee arthritis, fibromyalgia and other diseases. In 2000, Yocum suggested that Tai Chi might relieve pain and stress in rheumatoid arthritis patients, based on the link between stress and neuronal immune function (Yocum et al., 2000). The hypothesis that Chinese traditional exercises can alleviate musculoskeletal diseases was put forward. In 2003, Song conducted a randomized controlled trial study to explore the effects of Tai Chi on pain, balance, muscle strength in elderly women with osteoarthritis (Song et al., 2003). According to Astin’s research, mindfulness meditation combined with Qigong can alleviate pain and depression in patients with fibromyalgia. Astin et al. (2003) randomized controlled trial will provides high-level evidence for the effectiveness of traditional Chinese exercises for musculoskeletal disorders. In 2010, Wang CC published “A Randomized Trial of Tai Chi for Fibromyalgia” in The New England Journal of Medicine, a top medical journal, confirmed the therapeutic effect of Tai Chi on patients with fibromyalgia (Wang et al., 2010). This is the first article on traditional Chinese exercises published in the top journal in the medical field. High quality randomized controlled trial will promote research in the field, with the number of articles published in the following years more than doubling the number of articles published in 2010. In the subsequent study, besides Tai Chi, yi jin jing and baduanjin were also added to the research in this field. For example, An’s research in 2013 found that 1-year baduanjin is an effective and safe exercise method for the treatment of knee osteoarthritis (An et al., 2013). In 2011, Hall found that 10 weeks of Tai Chi practice improved pain and disability outcomes in patients with low back pain (Hall et al., 2011). In 2016, Lauche Romy’s study showed that 12 weeks of Tai Chi and traditional neck exercises were effective in improving neck pain and quality of life (Lauche et al., 2016). The number of diseases studied in this field has increased, such as low back pain, hip arthritis and neck pain. According to the keyword burst, neck pain was a hot research topic in this field from 2016 to 2018. In 2022, Kong published an article in Frontiers in Aging Neuroscience, which showed that traditional Chinese exercises significantly improved neck pain and disability in patients with neck pain and Baduanjin was the most effective (Kong et al., 2022). In recent years, there has also been a Chinese randomized controlled trial protocol on the intervention of neck pain with traditional exercise (Cheng et al., 2021). We suspect that neck pain may be a continuing research focus in the future.

Meta-analysis is a systematic review of quantitative analysis, which can synthesize the research results of multiple small samples on the same topic and improve the statistical efficiency of the original results. According to keywords burst systematic review and meta-analysis broke out from 2017 to 2020. Systematic review, published by Roger Chou in the annals of internal medicine in 2017, provides evidence that Tai Chi is effective for chronic low back pain, but the strength of the evidence is low (Rogers et al., 2010). Meta-analysis of comprehensive high-quality randomized controlled trials has been regarded as the highest level of evidence in evidence-based medicine, which can provide support for clinical guidelines. 2017 Qaseem incorporates Tai Chi into Non-invasive Treatments for Acute, Subacute, and Chronic Low Back Pain: Guideline from the American College of Physicians (Qaseem et al., 2017). The high level of clinical guidelines promote the clinical application of traditional Chinese techniques However, many Meta analysis points out that RCT in this field has shortcomings such as small sample size. Therefore, we speculate that on the basis of high-quality RCT studies in the future, the effectiveness of traditional Chinese exercises for musculoskeletal disorders will be further clarified.

Cluster two Keywords include Tai Chi; Exercise; Older Adult; Osteoarthritis; Quality of Life; Physical Activity; Balance; Muscle Strength. This cluster focused on the efficacy of traditional Chinese exercises for the treatment of musculoskeletal disorders dysfunctions. The main clinical manifestations of musculoskeletal disorders are pain and dysfunction, which seriously affect patients’ physical function (Balance and Muscle Strength) and quality of life. Traditional Chinese exercises can improve muscle strength, enhance balance and prevent falls. Hartman, published in the Journal of the American Geriatrics Society in 2000, has demonstrated that 12 weeks of Tai Chi can improve self-efficacy, quality of life indicators and functional activity in elderly patients with knee arthritis (Hartman et al., 2000). Studies have shown that Tai Chi can improve balance and abdominal strength by improving joint pain, stiffness and sensory difficulties. An’s study in 2008 found that Baduanjin can improve pain, stiffness and disability in patients with knee osteoarthritis and improve quadriceps strength (An et al., 2008). Runhaar’s 2015 study suggested that exercises including Tai Chi, increased thigh strength in patients with osteoarthritis, reduced stretching difficulties and improved proprioception (Runhaar et al., 2015). Song’s 2010 study found that 6 months of Tai Chi improved muscle strength around the knees, increased bone mineral density and reduced the fear of falling (Song et al., 2010). According to a systematic review in Age and Ageing, Tai Chi improves balance and fall risk in elderly people with knee arthritis (Mat et al., 2015). In 2020 Zhang’s found that the effect of Tai Chi on balance and fall prevention in patients with knee arthritis may be related to the enhancement of lower limb strength and reduction of foot load by Tai Chi (Zhang et al., 2020b). Tai Chi gait significantly increases external knee adduction moment and long-term practice may be harmful to patients with knee osteoarthritis (Simic et al., 2011). Therefore, Tai Chi gait is best used as an intermittent exercise for balance and stability, which can significantly improve the deep minor muscle group strength of knee flexors in the elderly. These studies are from the perspective of muscle strength and balance to explain the mechanism of traditional Chinese exercise in the treatment of knee osteoarthritis. According to the keyword burst, “prevention” is a hot research topic in this field from 2019 to 2022. The improvement of muscle strength and balance in Tai Chi is mainly explained in terms of clinical symptoms and biomechanics. Future studies may reveal the mechanism of Tai Chi in treating musculoskeletal and joint diseases from a biomechanical perspective.

Cluster three keywords included Pain; Knee Osteoarthritis; Management; Clinical Trials; Arthritis. This Cluster focused on Pain management for musculoskeletal disorders. Pain is an unpleasant sensory and emotional experience associated with actual or potential tissue damage or an experience similar to it. It is usually an important concomitant symptom of musculoskeletal disorders and profoundly affects the quality of life of patients. In 2009, Wang conducted a randomized controlled trial to explore the therapeutic effect of Tai Chi on knee osteoarthritis (KOA) and found that Tai Chi can not only effectively improve the physical function and quality of life of KOA patients, but also reduce the pain, anxiety and depression of KOA patients and improve self-efficacy (Wang et al., 2009). In 2016, it was confirmed that Tai Chi relieved KOA pain as much as standard physical therapy and showed greater improvement in quality of life and anxiety and depression than physical therapy (Wang et al., 2016). This evidence suggests that traditional Chinese exercises can play an important role as a mind-body therapy for the management of pain in musculoskeletal disorders.

Some researchers have explored the mechanism of Tai Chi in alleviating the pain of musculoskeletal disorders from neuroimaging. In 2019, Kong found that the effect of Tai Chi on pain improvement in fibromyalgia patients may be related to the enhancement of the cognitive control network and the resting-state functional connectivity of bilateral rostral anterior cingulate cortex (rACC)/medial prefrontal cortex (mPFC) (Kong et al., 2021). Chwan observed the changes of Tai Chi on KOA patient brain function by MRI and found that the improvement of Tai Chi on knee osteoarthritis pain pair was closely related to the increased resting-state functional magnetic resonance imaging connectivity observed between bilateral mPFC and amygdala seed regions (Shen et al., 2022). More studies have compared the specific changes of knee joint brain function with different Chinese traditional exercises. Studies have shown that both Tai Chi and Baduanjin can increase the resting state functional connectivity (rsFC) between supplementary motor area and bilateral dorsolateral prefrontal cortex (DLPFC), but there are differences in the changes of DLPFC rsFC between Tai Chi and Baduanjin (Shen et al., 2022). Current research into the mechanisms of musculoskeletal disorders has focused on knee arthritis. We believe that future studies will continue to reveal the specific effects of different traditional Chinese exercises on pain in musculoskeletal disorders from a neuroimaging perspective.

## Limitations

The limitations of the traditional Chinese exercises for musculoskeletal disorders study should be considered in further studies. First of all, our study did not search other academic databases and was limited to science Web, which may have missed some influential articles. Second, we aimed to study global trends in traditional Chinese exercises for musculoskeletal disorders, but the language range was limited. This may have led to selection bias. Third, this study includes articles and reviews, so there may be some bias in reflecting the academic impact of articles by the number of citations.

## Conclusion

We conducted a bibliometric analysis of the literature related to traditional Chinese exercises for musculoskeletal disorders over the past 22 years. We find a gradual increase in the number of annual publications, most of which appear in Medicine. The United States and China are the most published countries. The most relevant papers were published by Harvard Medical School, followed by Shanghai University of Traditional Chinese Medicine. But cooperation between different countries and institutions has been insufficient. Therefore, contact and communication should be strengthened to promote the application of traditional Chinese exercises for musculoskeletal disorders. According to the cited references and keywords, the main disease treated is osteoarthritis. At present, the focus of research in this field is still the safety and effectiveness of traditional Chinese exercises for the treatment of musculoskeletal disorders. At the same time, more research began to explore how traditional Chinese exercises played a role in the treatment of musculoskeletal disorders. Revealing the mechanism of traditional Chinese exercises from the interdisciplinary perspective of biomechanics and neuroimaging is the focus of research in this field and also the hotspot of future research.

## Data availability statement

The raw data supporting the conclusions of this article will be made available by the authors, without undue reservation.

## Author contributions

CG and FY conceived the idea. CG and YG collected the literature. CG, YG, and FX conducted the data analysis. CG drafted the manuscript. YG, FX, ZC, and FY revised the manuscript. All authors have read and approved the final article.
