# Supplementary material for: Global trends of traditional Chinese exercises for musculoskeletal disorders treatment research from 2000 to 2022: A bibliometric analysis
Source: Front Neurosci. 2023 Feb 10;17:1096789. doi: 10.3389/fnins.2023.1096789 (PMC9950260; doi:10.3389/fnins.2023.1096789)
Supplement: Supplementary file 1 [file Data_Sheet_1.docx]

Musculoskeletal Diseases #1

TS=(“Orthopedic Disorders”) OR TS=(“Orthopedic Disorder”) OR TS=(“Musculoskeletal Diseases”) OR TS=("Bone Diseases") OR TS=("Bone Cysts") OR TS=("Bone Diseases, Developmental") OR TS=("Bone Diseases, Endocrine") OR TS=("Bone Diseases, Infectious") OR TS=("Bone Diseases, Metabolic") OR TS=("Bone Malalignment") OR TS=("Bone Neoplasms") OR TS=("Bone Resorption") OR TS=("Coxa Magna") OR TS=("Coxa Valga") OR TS=("Eosinophilic Granuloma") OR TS=("Epiphyses, Slipped") OR TS=("Genu Valgum") OR TS=("Genu Varum") OR TS=("Hyperostosis") OR TS=("Osteitis") OR TS=("Osteitis Deformans") OR TS=("Osteoarthropathy, Primary Hypertrophic") OR TS=("Osteoarthropathy, Secondary Hypertrophic") OR TS=("Osteochondritis") OR TS=("Osteochondrosis") OR TS=("Osteonecrosis") OR TS=("Spinal Diseases") OR TS=("Cartilage Diseases") OR TS=("Chondromalacia Patellae") OR TS=("Laryngomalacia") OR TS=("Osteochondritis") OR TS=("Pectus Carinatum") OR TS=("Polychondritis, Relapsing") OR TS=("Tietze's Syndrome") OR TS=("Tracheobronchomalacia") OR TS=("Fasciitis") OR TS=("Fasciitis, Necrotizing") OR TS=("Fasciitis, Plantar") OR TS=("Foot Deformities") OR TS=("Foot Deformities, Acquired") OR TS=("Foot Deformities, Congenital") OR TS=("Hallux Valgus") OR TS=("Hallux Varus") OR TS=("Hammer Toe Syndrome") OR TS=("Metatarsal Valgus") OR TS=("Metatarsus Varus") OR TS=("Foot Diseases") OR TS=("Fasciitis, Plantar") OR TS=("Fibromatosis, Plantar") OR TS=("Heel Spur") OR TS=("Metatarsalgia") OR TS=("Posterior Tibial Tendon Dysfunction") OR TS=("Hand Deformities") OR TS=("Hand Deformities, Acquired") OR TS=("Hand Deformities, Congenital") OR TS=("Jaw Diseases") OR TS=("Bisphosphonate-Associated Osteonecrosis of the Jaw") OR TS=("Cherubism") OR TS=("Granuloma, Giant Cell") OR TS=("Jaw Abnormalities") OR TS=("Jaw Cysts") OR TS=("Jaw Neoplasms") OR TS=("Jaw, Edentulous") OR TS=("Mandibular Diseases") OR TS=("Maxillary Diseases") OR TS=("Joint Diseases") OR TS=("Ankylosis") OR TS=("Arthralgia") OR TS=("Arthritis") OR TS=("Arthrogryposis") OR TS=("Arthropathy, Neurogenic") OR TS=("Bursitis") OR TS=("Chondromatosis, Synovial") OR TS=("Contracture") OR TS=("Crystal Arthropathies") OR TS=("Femoracetabular Impingement") OR TS=("Hallux Limitus") OR TS=("Hallux Rigidus") OR TS=("Hemarthrosis") OR TS=("Hydrarthrosis") OR TS=("Joint Deformities, Acquired") OR TS=("Joint Dislocations") OR TS=("Joint Instability") OR TS=("Joint Loose Bodies") OR TS=("Metatarsalgia") OR TS=("Nail-Patella Syndrome") OR TS=("Osteoarthropathy, Primary Hypertrophic") OR TS=("Osteoarthropathy, Secondary Hypertrophic") OR TS=("Patellofemoral Pain Syndrome") OR TS=("Shoulder Impingement Syndrome") OR TS=("Synovitis") OR TS=("Temporomandibular Joint Disorders") OR TS=("Muscular Diseases") OR TS=("Arthrogryposis") OR TS=("Compartment Syndromes") OR TS=("Contracture") OR TS=("Craniomandibular Disorders") OR TS=("Eosinophilia-Myalgia Syndrome") OR TS=("Fatigue Syndrome, Chronic") OR TS=("Fibromyalgia") OR TS=("Isaacs Syndrome") OR TS=("Medial Tibial Stress Syndrome") OR TS=("Mitochondrial Myopathies") OR TS=("Muscle Cramp") OR TS=("Muscle Neoplasms") OR TS=("Muscle Rigidity") OR TS=("Muscle Spasticity") OR TS=("Muscle Weakness") OR TS=("Muscular Disorders, Atrophic") OR TS=("Musculoskeletal Pain") OR TS=("Myalgia") OR TS=("Myofascial Pain Syndromes") OR TS=("Myopathies, Structural, Congenital") OR TS=("Myositis") OR TS=("Myotonic Disorders") OR TS=("Myotoxicity") OR TS=("Paralyses, Familial Periodic") OR TS=("Polymyalgia Rheumatica") OR TS=("Rhabdomyolysis") OR TS=("Tendinopathy") OR TS=("Musculoskeletal Abnormalities") OR TS=("Arthrogryposis") OR TS=("Campomelic Dysplasia") OR TS=("Craniofacial Abnormalities") OR TS=("Developmental Dysplasia of the Hip") OR TS=("Funnel Chest") OR TS=("Gastroschisis") OR TS=("Klippel-Feil Syndrome") OR TS=("Limb Deformities, Congenital") OR TS=("Pectus Carinatum") OR TS=("Synostosis") OR TS=("Rheumatic Diseases") OR TS=("Arthritis, Juvenile") OR TS=("Arthritis, Rheumatoid") OR TS=("Fibromyalgia") OR TS=("Gout") OR TS=("Hyperostosis, Sternocostoclavicular") OR TS=("Osteoarthritis") OR TS=("Polymyalgia Rheumatica") OR TS=("Rheumatic Fever") OR TS=(“Back Pains”) OR TS=(“Pain, Back”) OR TS=(“Pains, Back”) OR TS=(“Backache”) OR TS=(“Backaches”) OR TS=(“Back Ache”) OR TS=(“Ache, Back”) OR TS=(“Aches, Back”) OR TS=(“Back Aches”) OR TS=(“Back Pain without Radiation”) OR TS=(“Vertebrogenic Pain Syndrome”) OR TS=(“Pain Syndrome, Vertebrogenic”) OR TS=(“Pain Syndromes, Vertebrogenic”) OR TS=(“Syndrome, Vertebrogenic Pain”) OR TS=(“Syndromes, Vertebrogenic Pain”) OR TS=(“Vertebrogenic Pain Syndromes”) OR TS=(“Back Pain with Radiation”) OR TS=(“Low Back Pain”) OR TS=(“Back Pain, Low”) OR TS=(“Back Pains, Low”) OR TS=(“Low Back Pains”) OR TS=(“Pain, Low Back”) OR TS=(“Pains, Low Back”) OR TS=(“Lumbago”) OR TS=(“Lower Back Pain”) OR TS=(“Back Pain, Lower”) OR TS=(“Back Pains, Lower”) OR TS=(“Lower Back Pains”) OR TS=(“Pain, Lower Back”) OR TS=(“Pains, Lower Back”) OR TS=(“Low Back Ache”) OR TS=(“Ache, Low Back”) OR TS=(“Aches, Low Back”) OR TS=(“Back Ache, Low”) OR TS=(“Back Aches, Low”) OR TS=(“Low Back Aches”) OR TS=(“Low Backache”) OR TS=(“Backache, Low”) OR TS=(“Backaches, Low”) OR TS=(“Low Backaches”) OR TS=(“Low Back Pain, Postural”) OR TS=(“Postural Low Back Pain”) OR TS=(“Low Back Pain, Posterior Compartment”) OR TS=(“Low Back Pain, Recurrent”) OR TS=(“Recurrent Low Back Pain”) OR TS=(“Low Back Pain, Mechanical”) OR TS=(“Mechanical Low Back Pain”) OR TS=(“Neck Pain”) OR TS=(“Neck Pains”) OR TS=(“Pain, Neck”) OR TS=(“Pains, Neck”) OR TS=(“Neck Ache”) OR TS=(“Ache, Neck”) OR TS=(“Aches, Neck”) OR TS=(“Neck Aches”) OR TS=(“Cervicalgia”) OR TS=(“Cervicalgias”) OR TS=(“Cervicodynia”) OR TS=(“Cervicodynias”) OR TS=(“Neckache”) OR TS=(“Neckaches”) OR TS=(“Cervical Pain”) OR TS=(“Cervical Pains”) OR TS=(“Pain, Cervical”) OR TS=(“Pains, Cervical”) OR TS=(“Posterior Cervical Pain”) OR TS=(“Cervical Pain, Posterior”) OR TS=(“Cervical Pains, Posterior”) OR TS=(“Pain, Posterior Cervical”) OR TS=(“Pains, Posterior Cervical”) OR TS=(“Posterior Cervical Pains”) OR TS=(“Posterior Neck Pain”) OR TS=(“Neck Pain, Posterior”) OR TS=(“Neck Pains, Posterior”) OR TS=(“Pain, Posterior Neck”) OR TS=(“Pains, Posterior Neck”) OR TS=(“Posterior Neck Pains”) OR TS=(“Anterior Cervical Pain”) OR TS=(“Anterior Cervical Pains”) OR TS=(“Cervical Pain, Anterior”) OR TS=(“Cervical Pains, Anterior”) OR TS=(“Pain, Anterior Cervical”) OR TS=(“Pains, Anterior Cervical”) OR TS=(“Anterior Neck Pain”) OR TS=(“Anterior Neck Pains”) OR TS=(“Neck Pain, Anterior”) OR TS=(“Neck Pains, Anterior”) OR TS=(“Pain, Anterior Neck”) OR TS=(“Pains, Anterior Neck”)

Traditional Chinese exercise #2

TS=(“Traditional Chinese exercise”) OR TS=(“Qigong”) OR TS=("Qi Gong”) OR TS=("Ch'i Kung”) OR TS=("Tai Ji”) OR TS=("Tai-ji”) OR TS=("Tai Chi”) OR TS=("Chi, Tai”) OR TS=("Tai Ji Quan”) OR TS=("Ji Quan, Tai”) OR TS=("Quan, Tai Ji”) OR TS=("Taiji”) OR TS=("Taijiquan”) OR TS=("T'ai Chi”) OR TS=("Tai Chi Chuan”) OR TS=(“yijinjing”) OR TS=(“Yi jin jing”) OR TS=(“Baduanjin”) OR TS=(“Ba duan jin”) OR TS=(“wuqiangxi”) OR TS=(“Wu qin xi”) OR TS=(“liuzikun”) OR TS=(“liu zi jue”)

#1 AND #2=#3

#4=(((#3) AND DOP=(2000-01-01/2022-08-14)) AND LA=(English)) AND DT=(Article OR Review)
